# Healthcare Process Modeling to Phenotype Clinician Behaviors for Exploiting the Signal Gain of Clinical Expertise (HPM-ExpertSignals): Development and evaluation of a conceptual framework

**DOI:** 10.1093/jamia/ocab006

**Published:** 2021-02-24

**Authors:** Sarah Collins Rossetti, Chris Knaplund, Dave Albers, Patricia C Dykes, Min Jeoung Kang, Tom Z Korach, Li Zhou, Kumiko Schnock, Jose Garcia, Jessica Schwartz, Li-Heng Fu, Jeffrey G Klann, Graham Lowenthal, Kenrick Cato

**Affiliations:** 1 Department of Biomedical Informatics, Columbia University, New York, New York, USA; 2 School of Nursing, Columbia University, New York, New York, USA; 3 Department of Pediatrics, University of Colorado Anschutz Medical Campus, Aurora, Colorado, USA; 4 Department of Medicine, Brigham and Women’s Hospital, Boston, Massachusetts, USA; 5 Department of Biomedical Informatics, Harvard Medical School, Boston, Massachusetts, USA

**Keywords:** electronic health records, predictive modeling, clinical informatics, conceptual framework

## Abstract

**Objective:**

There are signals of clinicians’ expert and knowledge-driven behaviors within clinical information systems (CIS) that can be exploited to support clinical prediction. Describe development of the Healthcare Process Modeling Framework to Phenotype Clinician Behaviors for Exploiting the Signal Gain of Clinical Expertise (HPM-ExpertSignals).

**Materials and Methods:**

We employed an iterative framework development approach that combined data-driven modeling and simulation testing to define and refine a process for phenotyping clinician behaviors. Our framework was developed and evaluated based on the Communicating Narrative Concerns Entered by Registered Nurses (CONCERN) predictive model to detect and leverage signals of clinician expertise for prediction of patient trajectories.

**Results:**

Seven themes—identified during development and simulation testing of the CONCERN model—informed framework development. The HPM-ExpertSignals conceptual framework includes a 3-step modeling technique: (1) identify patterns of clinical behaviors from user interaction with CIS; (2) interpret patterns as proxies of an individual’s decisions, knowledge, and expertise; and (3) use patterns in predictive models for associations with outcomes. The CONCERN model differentiated at risk patients earlier than other early warning scores, lending confidence to the HPM-ExpertSignals framework.

**Discussion:**

The HPM-ExpertSignals framework moves beyond transactional data analytics to model clinical knowledge, decision making, and CIS interactions, which can support predictive modeling with a focus on the rapid and frequent patient surveillance cycle.

**Conclusions:**

We propose this framework as an approach to embed clinicians’ knowledge-driven behaviors in predictions and inferences to facilitate capture of healthcare processes that are activated independently, and sometimes well before, physiological changes are apparent.

## INTRODUCTION

Clinical and physiological measurement data have limited predictive power for early detection of deterioration in hospitalized patients,^1^ prompting the search for additional, complementary predictive data sources. Clinicians are continuously engaged in complex decision making and pattern matching, informed by their knowledge and expertise, to drive skilled judgments (eg, prediction of patient trajectories) and subsequent actions (eg, increasing the frequency and type of clinical assessments needed).^2^ Measurement of the knowledge and expertise that drive clinical actions, particularly at scale, is inherently challenging. However, clinical actions are captured continuously through interactions with clinical information systems (CIS), including the electronic health record (EHR), medical devices (eg, intravenous pumps), and other healthcare equipment (eg, Pyxis); these actions provide an opportunity for scalable measures that can be exploited as signals of clinician knowledge and expertise (eg, the decision to order a test or further assess a patient).

System log files have been utilized to model patient states^3^ and can also be used to model clinician behavior patterns and workflows within and across institutions. Judgments and subsequent actions of clinicians may differ across individuals, specialties, settings, and expertise levels; yet, the care process is conducted in a consistent manner. The nursing process consistently drives nursing practice, is adapted from the scientific method, and is defined as the cyclical process: assess, diagnose, plan, intervene, and evaluate.^4^ Similar care process patterns drive the practice of other health professionals. While the correct action within each part of the care process may not always be executed (ie, care gaps and disparities exist), the care process is widely accepted and utilized across healthcare domains and can be used as an analytical framework.

## OBJECTIVE

To describe our development of the Healthcare Process Modeling Framework to Phenotype Clinician Behaviors for Exploiting the Signal Gain of Clinical Expertise (HPM-ExpertSignals). This framework illustrates (1) how to use CIS interactions to characterize behaviors, (2) how behavioral actions form proxies for clinical expertise and knowledge, and (3) how these proxies provide context that increase clinical predictive power of patient trajectories. This framework, which applies to all types of clinicians (eg, nurses, physicians, therapists), was developed based on our analytical approaches to phenotyping nurses’ behaviors related to clinical concerns to inform a predictive early warning score (EWS).^5^ While healthcare processes have been modeled previously,^6–8^ there has been no unified framework to date for systematically exploiting signals produced by the healthcare process in a way that can directly lead to changes in decisions and action at the bedside using clinical decision support (CDS).

## MATERIALS AND METHODS

**Figure 1.**
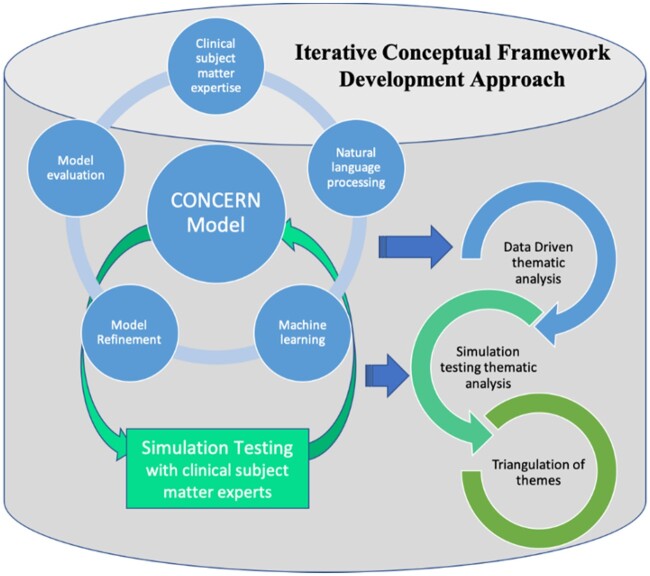
Approach to iterative conceptual framework development leveraging thematic analyses of processes and findings from data driven modeling and simulation testing for triangulation of themes. Thematic analysis of the iterative processes and contextual information that informed development of the Communicating Narrative Concerns Entered by Registered Nurses (CONCERN) model were triangulated with thematic analysis of clinical subject matter expert perceptions of the CONCERN model during simulation testing. These triangulated findings were used to define a conceptual framework for phenotyping clinician behaviors to detect and leverage signals of clinician expertise for prediction of patient trajectories.

We employed an iterative framework development approach that combined data-driven modeling and simulation testing with subject matter experts (SMEs) as part of the CONCERN (Communicating Narrative Concerns Entered by RNs) study (1R01NR016941-01), which includes 2 large academic medical centers in the northeastern United States. Our approach included iterative cycles of model refinement, engagement with SMEs, and triangulation of themes (see Figure 1). Institutional review board approval was obtained from both study sites.

### Data driven modeling use case: The CONCERN model

The CONCERN predictive model uses EHR data to identify signals of nurses’ concern that a hospitalized patient may be entering a risky state. The model is being implemented as an EWS in a user-centered designed CDS tool to foster communication and increase situational awareness among the interprofessional care team, particularly physicians and nurses. Nurses continuously monitor patients and document concerns throughout the EHR, such as in flowsheet comments; yet, this documentation is rarely viewed,^9–11^ despite evidence that a nurse’s concern is a valid reason to intervene without waiting for physiological changes.^2^^,^^12^^,^^13^ Unfortunately, if a nurse is concerned about a patient, there are a lack of visual functionality in the EHR that stand out in a similar way to visual cues used in a paper-based chart, such as “putting a note or colored paper in the front of the chart” or “circling concerning values in red” so that the chart or value can be readily identified among all the other patient charts or values. Our approach is to identify signals of equivalent types of behaviors in the EHR, validate which signals are predictive of patient trajectories, and surface those signals to the care team. A clinical trial will prospectively evaluate if the CONCERN CDS increases situational awareness among the care team and impacts outcomes (in-hospital mortality, length of stay, cardiac arrest, unanticipated transfer to the intensive care unit [ICU], and 30-day hospital readmission rates).

Other EWS use different approaches to identify at risk patients. The Modified Early Warning Score (MEWS),^14^ National Early Warning Score (NEWS),^15–18^ and the Rothman Index^19^^,^^20^ use changes in physiological data, such as abnormal vital signs, known to be late indicators of deterioration. The Rothman Index predicts death within 24 hours but not ICU transfer^21^—a clinically important marker of deterioration and an outcome in our study. The “worry factor”^22^ and the DENWIS (Dutch Early Nurse Worry Indicator Score)^23^^,^^24^ in their current state add to documentation burden. The CONCERN CDS does not require any new documentation or workflows by the nurse because our modeling approach uses behavior patterns to predict patient outcomes.

We achieve this by implicitly measuring expertise (nurse’s concern about patient status) and by explicitly measuring behavior-phenotypic differences between documentation patterns for deteriorating vs nondeteriorating patients.^5^ We incorporate information such as documentation patterns beyond standards of care and hospital requirements as environmental and system modifiers. Choosing to document beyond minimum requirements indicates a nurse likely determined an observed patient state was clinically significant enough to assess and record.^5^^,^^25^^,^^26^ This approach enables modeling when assessments and interventions are activated independent of standards of care or documentation requirements to infer that the nurse’s decision making reflected an increased level of concern.

We used nurses’ EHR interaction data, which are high volume, curated using the standardized ontology that we developed within the i2b2 (Informatics for Integrating Biology and the Bedside; https://www.i2b2.org) system of all inpatient encounter information, including patient demographics, flowsheets, notes, diagnoses, significant events, laboratory tests, orders, medication administrations, clinician information, and admission, discharge, and transfer information.^27^^,^^28^ We previously published about this sharable ontology, which leverages the Unified Medical Language System, the Clinical Care Classification System, and nurse SME validation.^29^^,^^30^ This ontology is the conceptual data model for the CONCERN study’s databases of hospitalizations from 2015 to 2018 (site A = 123 981 patients and 697 928 178 data points; site B = 188 512 patients and 191 904 328 data points). The 2 sites each used a different EHR, accounting for the wide range of data points generated per patient.

We targeted general medical-surgical acute care units, intermediate care (ie, stepdown units), and intensive care units (ie, critical care) to analyze data for patients that spent more than 24 hours on a study unit, excluding patients younger than 18 years of age, receiving hospice and palliative care, or with a hospital stay exceeding 60 days.^14^

We capture metadata associated with each structured clinical data point, such as unique patient and hospital visit identifiers and timestamps. Our analyses focused on the number of entries (not the value, but rather the frequencies of data entry such as vital signs, notes, or comments). Model refinement used longitudinal logistic and hazard regressions with time-varying covariates.^31–33^ Our data-driven analyses includes natural language processing of unstructured, narrative data from nursing notes and free-text comments.^30^^,^^34^ Methods for data cleaning and validation are previously reported,^5^^,^^34^^,^^35^ as are the development and performance of CONCERNv1.0 model.^5^ We report on the CONCERNv2.0 model in this study. The output of the CONCERNv2.0 model was translated into a categorical score of red (high risk), yellow (moderate risk), and green (low risk) based on user specified feedback, reduction of score variability, and corrections for demographic biases. A description of our score development will be published separately.

We measured the performance of the CONCERNv2.0 model by using the Cox time-varying proportional hazards model with our composite outcome as the endpoint, defined as the first occurrence of in-hospital mortality, cardiac arrest, unanticipated transfer to the intensive care unit, rapid response, or sepsis. We selected the well-published MEWS and NEWS as comparators because they are based solely on a patient’s physiological data and are the most widely used and validated EWS models.^14–18^ Specifically, we used as a feature set each patient’s hourly CONCERN score (low = green, moderate = yellow, high = red), MEWS score (low = 0-2, moderate = 3-4, high = 5+), and NEWS score (low = 0-3, moderate = 4-6, high = 7+) to quantify whether the presence of an increasing score predicted our composite outcome. The thresholds used to determine low, moderate, and high risk for MEWS and NEWS are described elsewhere.^36^^,^^37^ Further, as part of our evaluation, we constructed a model using the CONCERN, MEWS, and NEWS scores, with low risk scores as the control. We also computed a “lead time” statistic which describes how well an observed risk level differentiates between events and nonevents at various hours in the future. The statistic is based on the likelihood ratio of the 2 probability measures induced by events and nonevents and is a natural choice for sequential hypothesis testing.

### Thematic analysis from development of the CONCERN model

Thematic analysis from our data-driven modeling was performed by Sarah Collins Rossetti by analyzing the processes and contextual information that informed feature selection and grouping of the types of features that emerged. Group sessions were conducted during team meetings to achieve consensus related to the identified themes.

### Thematic analysis from simulation testing of the CONCERN model

We conducted simulation studies with nurses and physicians to understand their perspective of the CONCERN model and CDS tool using the Situation Awareness Global Assessment Technique method.^38^ CDS prototypes^5^ were presented to 17 nurses and 6 physicians as part of 6 simulated patient cases with varied risk levels to evaluate impact on situational awareness and provide user-centered design feedback. Data were analyzed by Sarah Collins Rossetti, Min Jeoung Kang, and GL for themes related to nurses’ and physicians’ perspectives of the model. Group sessions were conducted during team meetings to achieve consensus related to the identified themes.

### Triangulation of themes for iterative conceptual framework development

Themes identified from our data-driven modeling and simulation testing were triangulated to define a conceptual framework for phenotyping clinician behaviors to detect and leverage signals of clinician expertise for prediction of patient trajectories (see Figure 1). Specifically, the themes were compared and evaluated to understand how they align with each other and were integrated into a conceptual framework by Sarah Collins Rossetti with group consensus sessions for refinements conducted during team meetings.

## RESULTS

### Data driven modeling use case: The CONCERN model

The CONCERNv2.0 model included 15 measurement features, which were expanded to 30 measurement features based on whether they were measured at common or uncommon times, 21 nursing note content features, and 4 temporal features (Table 1). The integration of additional data types (eg, demographics, location in hospital, time features) were modifiers that enriched predictive power.

**Table 1.** CONCERN model features

Features			
Measurements and Temporal	Clustered^a^	Note Content	Clustered^a^
Heart rate measurement^b^	Yes	Abdominal pain^b^	No
Respiratory rate measurement^b^	Yes	Abnormal heart rhythm^b^	No
Blood pressure measurement^b^	Yes	Abnormal mental state^b^	No
Temperature measurement^b^	Yes	Abnormal rate, rhythm, depth and effort of respirations^b^	No
SpO_2_ measurement^b^	Yes	Abnormal temperature^b^	No
All 5 vital measurements taken at same time^b^	Yes	Back pain^b^	No
Only 1 vital measurement taken^b^	Yes	Chest pain^b^	No
Heart rate comment^b^	Yes	Communication problem^b^	No
Respiratory rate comment^b^	Yes	Diagnosis related with infection^b^	No
Blood pressure comment^b^	Yes	Deficit of circulation^b^	No
Temperature comment^b^	Yes	Fall risk^b^	No
SpO_2_ comment^b^	Yes	Fluid volume alteration^b^	No
PRN medication administered^b^	Yes	General concern^b^	No
Scheduled medication withheld^b^	Yes	Headache^b^	No
Nursing note written^b^	Yes	Improper renal function^b^	No
Month	No	Medication related with infection^b^	No
Day of week	No	Monitoring^b^	No
Hour	No	Mood disorder^b^	No
Patient Hour	No	Musculoskeletal pain^b^	No
		Pain level^b^	No
		Violence gesture^b^	No

CONCERN: Communicating Narrative Concerns Entered by Registered Nurses; PRN: as needed; SpO_2_: oxygen saturation.

a Whether the feature is clustered into times it is commonly measured or uncommonly measured.

b Feature is aggregated over the past 12 hours.

**Figure 2.**
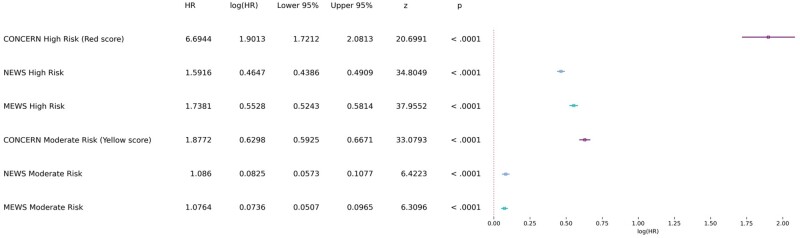
Time-varying survival regression. Forest plot of the covariates used in Cox time-varying proportional hazards model and associated statistics. HR: hazard ratio; MEWS: Modified Early Warning Score; NEWS: National Early Warning Score.

**Figure 3.**
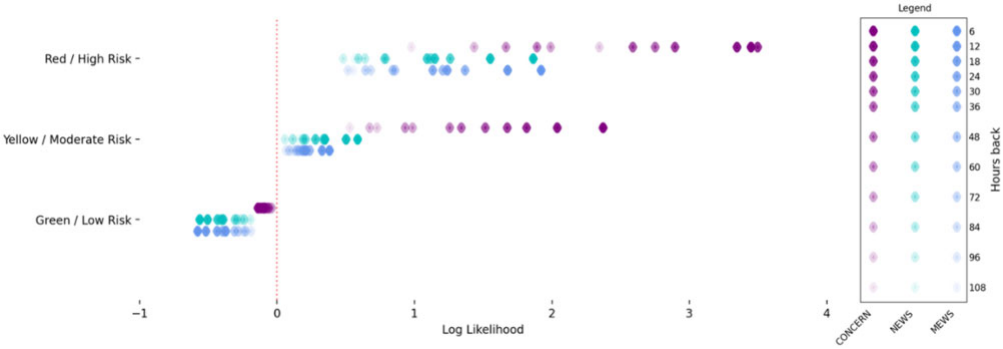
Comparison of log likelihood ratios at various hours before event. The likelihood ratio, defined as L(*x,h*) = P(*x* | patient has an event *h* hours in the future) / P(*x* | patient does not have an event *h* hours in the future). For example, L(‘CONCERN score = yellow’, 6) quantifies how well the Communicating Narrative Concerns Entered by Registered Nurses (CONCERN) algorithm separate the probability measures induced by whether the patient has an event 6 hours in the future after observing a “yellow” score. Larger values represent more weight given to the numerator vs the denominator, while smaller values represent more weight given to the denominator. MEWS: Modified Early Warning Score; NEWS: National Early Warning Score.

Our model evaluation, using the Cox time-varying proportional hazards model, showed that, with CONCERN low risk as the control, the hazard ratio for CONCERN moderate risk is 3.42 (95% confidence interval, 3.28-3.57; *P <* .0001), meaning that a moderate risk implies 2.42 greater hazard of cardiac arrest above low risk. Similarly, the hazard ratio for CONCERN high risk is 13.32 (95% confidence interval, 11.02-16.1; *P <* .0001), meaning that a high risk implies 12.32 greater hazard of cardiac arrest above low risk. In our model using CONCERN, MEWS, and NEWS low risk scores as the control, the CONCERN high risk score implied greater hazard than both the MEWS and NEWS high risk scores (6.69 vs 1.74 and 1.59). The CONCERN moderate risk score also implied greater hazard than both the MEWS and the NEWS moderate risk scores (1.88 vs 1.08 and 1.09) (see Figure 2). All 4 comparisons (CONCERN high vs NEWS high; CONCERN high vs MEWS high; CONCERN moderate vs NEWS moderate; CONCERN moderate vs MEWS moderate) are significant at the *P <* .0001 level. We also demonstrated that the CONCERNv2.0 model had a better “lead time” compared with MEWS and NEWS in the sense that CONCERN was able to better differentiate between a patient’s probability of an event earlier (see Figure 3). For instance, the likelihood of an event occurring 48 hours after observing a CONCERN high risk score is comparable to the likelihood of an event occurring 6 hours after observing a high risk MEWS or NEWS score—a difference of 42 hours.

### Themes from development of the CONCERN model

We identified 3 themes described subsequently based on our findings from our approach to developing the CONCERN model.

#### Theme 1: Predictive signals may be derived from clinical behaviors

The first theme was that user interaction data contain patterns of clinician behaviors that can be interpreted as proxies of an individual’s decisions, knowledge, and expertise and used in predictive models and visualizations for associations with outcomes. This theme is supported in our CONCERNv2.0 model features, such as “documentation at uncommon times” and “withheld scheduled medication.” These types of actions reflect nursing decisions that are consistent with increased clinical concern and can predict patients’ states. The predictive signals are derived from analysis of complex patterns of clinician behaviors (ie, behavioral phenotypes),^39^ which are distilled and displayed to clinicians in the CONCERN CDS. These novel patterns identified through machine learning are in contrast to MEWS and NEWS that use physiological data thresholds (eg, abnormal vital signs) that are already applied by clinicians as part of routine clinical decision making.

#### Theme 2: Clinical domain expertise are essential for interpretations

The second theme is that clinical domain expertise is essential for accurate and comprehensive data integrations and interpretations. For example, in investigating our findings, we identified that the frequency of documenting respiratory rate and oxygen saturation (SpO_2_) comments consistently had a strong signal. We previously highlighted how understanding that respiratory rate is the only vital sign manually measured in the hospital setting for nonventilated patients and provides important contextual information when interpreting the frequency with which it is documented.^5^ We also previously reported how nurses explained that SpO_2_ comments are typically used to highlight to the physician when the nurse continues to titrate the supplemental oxygen in order to keep the SpO_2_ reading normal for the patient—an indicator of deteriorating status that can be lost among EHR data points.^11^

#### Theme 3: Temporal focus drives clinical utility

The third theme is that time-based features, and other types of modifiers, drive utility of healthcare process modeling in the clinical setting. Most EWS models use vital sign abnormalities to predict deterioration but have shown limited impact on clinical outcomes because vital signs are a late indicator of deterioration.^1^^,^^40^^,^^41^ The CONCERN CDS tool aims to surface early changes in a patient state for the care team’s situational awareness of patient risk. In order to do so, we identify when nurses were concerned that a patient may be entering a risky state—as opposed to when physiological values indicate they are already in a risky state—by modeling with a simulated real-time prospective analytical approach that accounts for the temporal signal of each data point.

The themes described previously are aligned with an approach to feature selection based on user interaction with the machinery that supports and captures the timely delivery of care and clinical interventions (eg, EHR data/metadata, smart pumps, communication devices) and is informed by clinical domain knowledge.

### Themes from simulation testing of the CONCERN model

We also identified 4 themes from the simulation study, each with 2 to 4 subthemes. The 4 themes were clinical decision making, paradigm shift, believability, and CIS interactions. Subthemes were coded with positive and negative perspectives as shown in Table 2.

Identified themes demonstrated the relevance of the CONCERN model from the perspective of practicing clinicians. The perceived benefits to clinical decision-making were positive, including improved prioritization, team-based communication, critical thinking, and EHR information overload. Clinicians recognized the important paradigm shift and potential in detecting features not readily observable from physiological measures, while acknowledging dissemination challenges for a prediction model in the clinical setting that does not rely on physiological values.^42^^,^^43^ The model was believable by clinicians, with several noting that it reflected their observed practice patterns. However, some noted that “back-charting” (ie, data entered retrospectively in-batch) may limit real-time CDS interventions (although back-charting can be computationally detected). Some cautioned that a system-derived model may further decrease face-to-face patient time; yet, others recognized the utility in driving more efficient patient prioritization through rank ordering patients by risk level.

### Triangulated themes and HPM-ExpertSignals conceptual framework development

**Figure 4.**
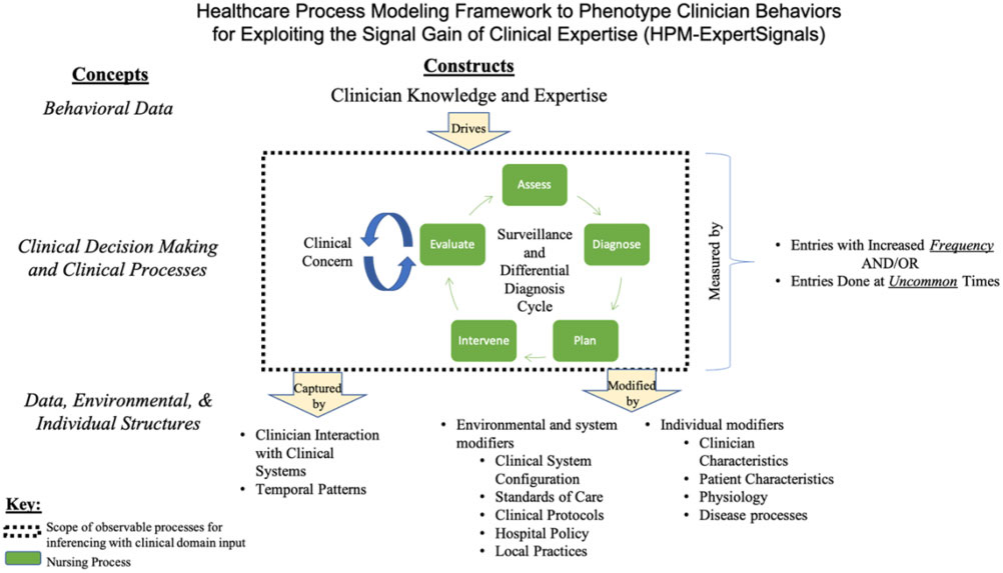
Healthcare Process Modeling Framework to Phenotype Clinician Behaviors for Exploiting the Signal Gain of Clinical Expertise (HPM-ExpertSignals). The framework is focused on information that can be mined from clinical data structures, is generated by clinician processes, and is driven by knowledge-based behaviors in order to identify features from user interaction with clinical systems, which are patterns of clinical behaviors and can be interpreted and used in predictions.

Figure 4 conveys our HPM-ExpertSignals Conceptual Framework, which leverages an adaptation of Donabedian’s structure-process-outcome framework, ^44^ and is focused on information that can be mined from clinical data structures, generated by clinician processes, and driven by knowledge-based behaviors. The developed framework includes a 3**-**step modeling technique to phenotype clinician behaviors as proxies of clinician knowledge and expertise to inform predictive models: (1) identify features from user interaction with clinical systems that are patterns of clinical behaviors; (2) interpret patterns as proxies of an individual’s decisions, knowledge, and expertise; and (3) use patterns in predictive models for associations with outcomes. We found that when observing patterns of clinical activity and behavior, domain experts interpret those patterns in the context of clinical knowledge and expertise, deducing the sources of those behaviors. The framework conveys that by observing clinician activity pattern distributions for subgroups of patients, we can quantify and systemize the observed behavioral expertise such that it can be leveraged to predict patient trajectories. Based on our iterative data-driven modeling and simulation testing with SMEs, we identified 4 main challenges in measuring clinician expertise and 4 main challenges in analyzing CIS interactions Table 3). These challenges are surmountable when informed by appropriate domain expertise, data**-**driven methodologies, and awareness of the limitations for inferencing.

**Table 2.** Themes derived through CONCERN model simulation testing

Theme	Subtheme
Clinical decision making	(+) Use of model drives improved critical thinking
	(+) Use of model drives improved patient prioritization
	(+) Use of model drives improved team-based care and communication
	(+) Features from the model synthesize the chart to decrease information overload
Paradigm shift	(+) Model is an innovative method for recognizing increased patient risk above baseline
	(-) Model does not use clinical data values
Believability	(+) Model’s concerning surveillance patterns reflect clinical practice
	(+) Model’s use of nursing documentation validates the value of nurses’ documentation efforts
CIS interactions	(-) Missing and back-charted EHR data may limit predictions
	(-) Model’s focus on CIS interactions may perpetuate an over-reliance on clinical systems and decreased patient interactions

CIS: clinical information systems; CONCERN: Communicating Narrative Concerns Entered by Registered Nurses; EHR: electronic health record.

(+) indicates a positively perceived theme by simulation testing participants and (-) indicates a negatively perceived theme by simulation testing participants.

**Table 3.** Main challenges in measuring clinician expertise and analyzing CIS interactions

Challenges in measuring clinician expertise
1. Expert judgments may derive from unconscious or unrecorded observations.
2. Experts are often unable to articulate guiding cues.^45^^,^^46^
3. Differentiating expert-driven actions from inexperienced actions is not always possible.
4. Expertise changes overtime and is context dependent.
Challenges in analyzing CIS interactions
1. Health professionals’ care processes include shared *and* divergent activities and reasoning, requiring appropriate domain knowledge to identify and interpret.
2. Factors that modify system interactions (eg, configurations, standards of care, policies) vary within and across institutions.
3. Individuals do not always behave rationally—noise and diversity exist among the consistency of clinical processes.
4. All possible actions and best practices are not known or captured.

CIS: clinical information systems.

Several factors modify CISs interactions (see Figure 4). At the individual level, clinician characteristics (eg, experience, patient load), patient characteristics (eg, acuity, code status), and physiological and disease processes may modify CIS interactions. Environmental and system modifiers also impact CIS interactions, such as system configurations (eg, hard stops, documentation by exception), standards of care, policy or regulatory requirements, and protocols, all of which may vary by setting (eg, academic medical center, rural hospital) or specialty. Some factors are challenging to explicitly incorporate, including local environmental factors, such as clinical standards adherence, variable clinical roles and responsibilities, and specialist availability. Traditional predictive modeling can detect some environmental and system modifiers through latent factors, but HPM-ExpertSignals encourage explicit modeling of difficult-to-detect modifiers using a top-down modeling approach.^6^^,^^47^^,^^48^ For example, our team has demonstrated the implicit identification of contextual factors and data-driven covariates for modeling laboratory data^47^ and survival models of chronic kidney disease^48^ for healthcare process biases and effects.

## DISCUSSION

HPM-ExpertSignals moves beyond transactional data analytics to model clinical knowledge, decision making, and behavior to make predictions about patients whose risk is unexpectedly higher than it appears from physiological data alone, in contrast to patients whose risk state is obvious to clinicians based on abnormal data values. An evaluation of the CONCERN model, or any EWS model, based on clinical outcomes may underrepresent its impact because some “at-risk” patients are intervened on in the clinical setting and therefore do not experience a negative outcome. The CONCERN use case verifies our ability to phenotype nursing behaviors, associate behaviors to outcomes, and use those associations to make actionable predictions. This approach to health data modeling, broadly, can advance how we understand clinical observational skills and clinician-entered data, and can inform phenotyping studies.

“Surveillance” is characterized within the nursing process by the rapid and frequent cycle of assess, diagnose, plan, intervene, and evaluate, and includes the iterative relationship between decision making and care processes. In applying these concepts, the CONCERN model captured when (1) nurses change surveillance and interventions patterns (eg, PRN [as needed] medications administered, scheduled medications withheld) and documentation frequency and (2) nurses use notes and short narrative comments to contextualize and highlight specific flowsheet values. These concerning patterns include features consistent with implicit (eg, increased number of documentation entries during uncommon times) and explicit (eg, EHR comments to highlight inadequate physiological response) models of expertise. Used in context, a nurse may convey increased surveillance of SpO_2_ and supplemental oxygen by entering these data more frequently, and use comments associated with those structured data to annotate and highlight the patient’s inadequate SpO_2_ response to increased supplemental oxygen.^25^ In other words, documentation patterns contain predictive signals that can be detected before signals that are derived from the data values within documentation. Increased streaming of continuous monitoring data into EHRs will decrease manual data entries; however, nurses will likely increase annotations and highlighting of data. Annotation and highlighting of data are the types of EHR workflows HPM-ExpertSignals targets. The CONCERN model will be adapted to new EHR workflows as they emerge.

Our prior work confirmed nurses’ intentions to highlight deteriorating status to physicians.^25^ Patient deterioration is a complex patient safety problem with dependencies on effective team communication and intervention activation.^2^^,^^24^^,^^49^^,^^50^ In the CONCERN example, the nurse is already aware that the patient is at risk. The CONCERN CDS surfaces predictions of nurses’ concern so that they are visible to the care team.

Our model development demonstrated improvement when inferences on EHR data were informed by clinical knowledge, data generation processes, and clinical practice; omitting these inputs is usually negative. For example, medication administration data have patterns and signals associated with outcomes that are distinct from medication orders^27^^,^^51^; when interpretation is not informed by clinical knowledge, medication administration data might be assumed to be redundant with orders, potentially leading to inaccurate assumptions.^52^ In contrast to unsupervised approaches, which miss the signal gain of clinical expertise,^52^ HPM-ExpertSignals provides a framework for making clinical domain knowledge inferences explicit. For example, analyses of flowsheet^53^ and medication data^51^ must account for workarounds and the shift-based nature of nursing work to overcome data incompleteness and timestamp issues that may not be apparent to nondomain experts.^11^^,^^25^^,^^51^^,^^53^ Phenotyping studies in the absence of HPM-ExpertSignals may miss the greater context of clinical expertise, workflows, and decision making (eg, differential diagnoses, nursing surveillance) driving data capture and the characteristics of clinical data being used to define phenotypes.^54–60^

Features that are proxies of clinical judgment are, by definition, interpretable and may advance predictive power.^2^^,^^61^ Clinical observations and clinician-entered data, when analyzed in the context of standards of care and policy or regulatory requirements, provide insight into the decision-making processes that drive data capture.^54–60^ To accelerate phenotyping of clinician behaviors, we recommend that common data models incorporate additional data and metadata (eg, user, time of day, system configuration) of CIS interactions, including EHR flowsheets. Further, phenotypes of system interactions and clinician-entered data could expose problematic documentation burden patterns.

Future work includes application and validation of the HPM-ExpertSignals framework to use cases in other clinical domains, such as concern for patients’ self-management capability or identifying implicit and explicit biases linked to outcome disparities.^62^ Characterizing system interactions from a team perspective, and as potential intervention points for CDS or decreasing documentation burden, are also additional future research areas. Future methodological advancements include approaches to handle temporal data and broader incorporation of healthcare process variables, including physiology.

## CONCLUSION

In this study our CONCERN model use case demonstrated that focusing only on clinical values may miss healthcare processes and interventions that are activated independently, and in some cases, long before physiological changes are apparent. Physiological values contain different information than documentation patterns. We developed the HPM-ExpertSignals framework as an approach to predict information that cannot be inferred from physiological values. Phenotyping clinician behaviors through characterizations of system interactions may advance how we characterize and utilize clinical observations and clinician-entered data for knowledge generation and modeling of decision making, and to embed clinician knowledge in predictions.

Health data volume increases every day; our HPM-ExpertSignals framework illustrates that the value trapped within these data extends far beyond explicitly recorded physiological values and treatments. Novel signals can be exploited to provide more information than is present in explicit data elements alone.^5^^,^^26^^,^^42^^,^^63^ CIS metadata are widely underutilized and our work demonstrates that these data contain unique, implicit behavioral markers of bedside observation and decisions about care intensity that cannot be acquired through other data collection methods. Understanding clinicians’ choice to interact with CIS, and how those choices impact data signals or statistical biases, is essential in predictive modeling.

## FUNDING

This work was supported by the National Institute of Nursing Research: 1R01NR016941-01, Communicating Narrative Concerns Entered by RNs (CONCERN): Clinical Decision Support Communication for Risky Patient States and the National Institute of Nursing Research Reducing Health Disparities Through Informatics T32NR007969. The content is solely the responsibility of the authors and does not necessarily represent the official views of the National Institutes of Health.

## AUTHOR CONTRIBUTIONS

SCR and KC conceived and designed the study. All authors contributed to collection and analysis of the data. SCR drafted the manuscript, and all authors contributed substantially to its revision. SCR and KC take responsibility for the paper as a whole.

## COMPETING INTERESTS STATEMENT

The authors have no competing interests to declare.

## DATA AVAILABILITY STATEMENT

The data underlying this article were accessed from Columbia University Irving Medical Center and Mass General Brigham. The derived data generated in this research will be shared on reasonable request to the corresponding author.
